# The Resignation of Dr. Love

**Published:** 1896-09

**Authors:** 


### THE RESIGNATION OF DR. LOVE

DR. I. N. LOVE, of St. Louis, as stated in another column, has resigned the chair of clinical medicine and diseases of children that he has held since the Marion-Sims college of medicine was founded.

Dr. Love is an original thinker and a forceful speaker. As a teacher his influence was weighty, and he will be missed in the college where his popularity was great and deserved.

In the *Medical Mirror* for July, 1896, Dr. Love comments as follows on his separation from the college :

The editor of this journal, after having served as a member of the faculty of the Marion-Sims College of Medicine from the beginning, being one of the original charter members of the faculty ; having seen the college develop until it is now recognised as one of the leading medical colleges of the West and South, has tendered his resignation.

He has felt for two or three years past that his duty to himself and his own best interests lay in this direction, but a hesitancy in withdrawing from pleasant association with his colleagues until the success of the institution was secure, has postponed this action. In his letter of resignation he expressed his regrets and a warm personal regard for the faculty individually and collectively, and announced that his private practice, connected with his conduct of the *Medical Mirror*, was such as to demand a more complete concentration than was possible by a continuance of college work.

The resignation was accepted, accompanied by expressions of appreciation and good wishes. And now, being relieved to a degree of the inconvenience of having too many irons in the fire, the editor feels that the *Medical Mirror* will be the gainer.

When he established the *Medical Mirror* six years ago he announced that he had started upon a life-work, and this determination has been strengthened as time has passed. He is ambitious to make the *Medical Mirror* not only one of the best medical magazines in the world, but to have it read by more doctors than any other.

Plans that are under way now will soon be consummated in the direction of the accomplishment of this end. In saying good-bye to the Marion-Sims college of medicine and its faculty, the editor of the *Mirror* desires to express, in most emphatic manner, the hope that, as the years come and go, the banner of the Marion-Sims college may never be furled ; that her reputation may go on and on ringing round the world, a crystallisation of higher medical education and the best good of the profession. To the faculty he would say, individually and collectively, May the Lord love you and not call you too soon.

---

THE Convalescent Dinner Society is a London association which undertakes the duty of granting in well-authenticated cases, in which sickness has reduced the strength necessary to return to work, an order for fourteen daily dinners. Such orders have been granted to nearly one-thousand convalescents during the last year.—*Chicago Medical Recorder.*

